# Recent Progress in Cardiovascular Research Involving Single-Cell Omics Approaches

**DOI:** 10.3389/fcvm.2021.783398

**Published:** 2021-12-16

**Authors:** Zhehao Dai, Seitaro Nomura

**Affiliations:** Department of Cardiovascular Medicine, Graduate School of Medicine, The University of Tokyo, Tokyo, Japan

**Keywords:** single-cell omics, cardiovascular research, heart development, cardiac homeostasis, myocardial infarction, heart failure

## Abstract

Cardiovascular diseases are among the leading causes of morbidity and mortality worldwide. Although the spectrum of the heart from development to disease has long been studied, it remains largely enigmatic. The emergence of single-cell omics technologies has provided a powerful toolbox for defining cell heterogeneity, unraveling previously unknown pathways, and revealing intercellular communications, thereby boosting biomedical research and obtaining numerous novel findings over the last 7 years. Not only cell atlases of normal and developing hearts that provided substantial research resources, but also some important findings regarding cell-type-specific disease gene program, could never have been established without single-cell omics technologies. Herein, we briefly describe the latest technological advances in single-cell omics and summarize the major findings achieved by such approaches, with a focus on development and homeostasis of the heart, myocardial infarction, and heart failure.

## Introduction

Cardiovascular diseases are among the major causes of morbidity and mortality worldwide. Yet, despite relentless research efforts in recent decades, the complex structure of the heart, its incessantly beating nature, and its wide range of abnormalities and disorders made us still struggle to understand the molecular mechanisms underlying the normal development, homeostasis, and diseases of this organ.

**Figure 1:**
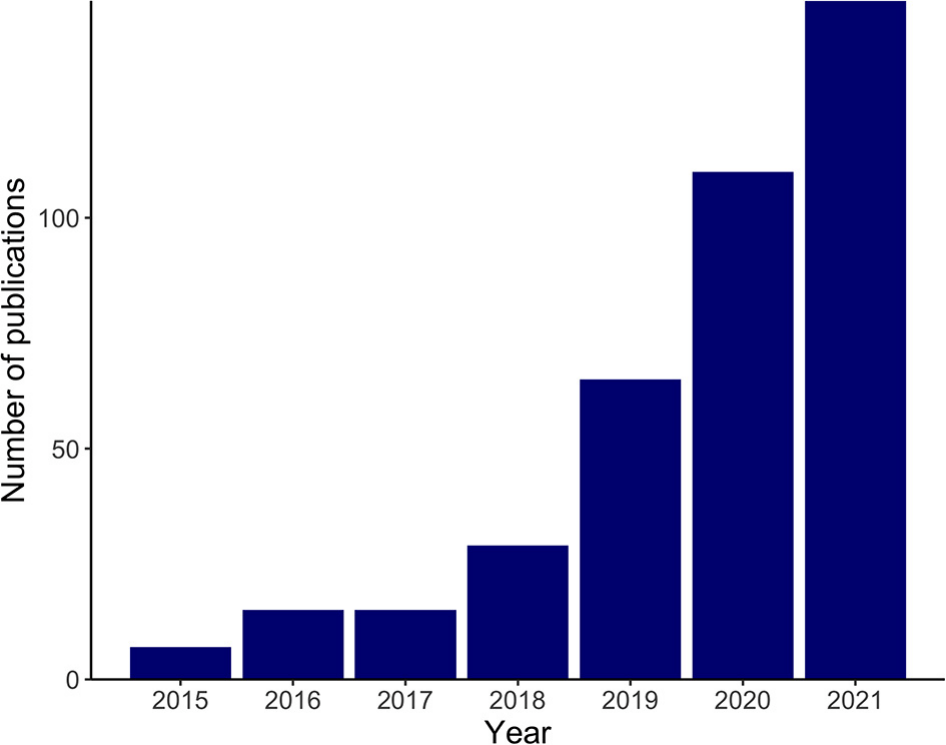
Exponential increase in cardiovascular research implementing single-cell omics. The bar plot shows the numbers of search results in PubMed in recent years with the following search term and the search conducted on November 2, 2021: (“RNA-Seq”[Mesh] OR “Transcriptome”[Mesh] OR “Chromatin Immunoprecipitation Sequencing”[Mesh] OR multi-omics[Title/Abstract] OR multiomics[Title/Abstract] OR omics[Title/Abstract] OR sequencing[Title/Abstract] OR seq[Title/Abstract]) AND (“Single-Cell Analysis”[Mesh] OR “single cell”[Title/Abstract] OR “single nucleus”[Title/Abstract] OR “single nuclei”[Title/Abstract]) AND (“heart”[Mesh] OR heart[Title/Abstract] OR cardiac[Title/Abstract] OR cardiomyocyte[Title/Abstract] OR myocardial[Title/Abstract]).

Recent technological advances have enabled researchers to understand biology at the single-cell resolution. Single-cell RNA sequencing (scRNA-seq) and single-cell assay for transposase-accessible chromatin sequencing (scATAC-seq) allow an unbiased assessment of transcriptomics and epigenomics in heterogeneous tissues and have identified not only previously unknown cell populations, but also dynamic cellular transitions and intercellular interactions in various tissues (1, 2). These cutting-edge technologies have been applied not only to healthy humans and mice to create reference maps, namely, the Human Cell Atlas and the Tabula Muris (3, 4), but also to normal development and various diseases. Recent advances in spatial transcriptomics, the molecular profiling method that compensates for the lack of spatial information in scRNA-seq, have furthered our understanding (5). The use of single-cell omics technologies in cardiovascular research has been increasing exponentially since 2015 (Figure 1) and, as expected, is providing more insight into the workings of both healthy and pathological hearts.

In this review, we briefly introduce the latest technological advances in single-cell omics and summarize recent progress in cardiovascular research that has applied these technologies, with special focus on development and homeostasis of the heart, myocardial infarction (MI) and ischemia, and heart failure (HF). We also discuss how single-cell omics can, and should support future cardiovascular research and contribute to precision cardiovascular medicine.

## Technological Advances

The experimental pipelines of single-cell omics, represented by scRNA-seq, typically begin with single-cell isolation and capture, followed by sequencing and downstream analyses (6). In the early days, integrated fluidic circuit (IFC) systems, such as Fluidigm C1, were used to isolate cells into individual chambers, where they were then lysed and reverse-transcribed (plate-based method). The IFC system has been largely replaced by fluorescence-activated cell sorting (FACS), which can not only isolate cells, but also distinguish live cells through viability stains. A major drawback of plate-based systems, however, is the limited number of cells that can be analyzed. To overcome this, droplet-based methods were developed, in which single cells are encapsulated in droplets and then undergo barcoding and reverse transcription. This technology has greatly improved throughput, increasing the number of cells analyzed to up to 10,000 cells, albeit with a sacrifice in read depth.

Both IFC systems used in plate-based methods and microfluidic devices used in droplet-based methods limit the size of the cells that can be processed to < ~40 μm, which are applicable to non-cardiomyocytes, neonate cardiomyocytes (CMs), and pluripotent stem cell-derived CMs. However, adult CMs (>100 μm in length) cannot be processed by these methods. Moreover, commercially available FACS nozzles (70–130 μm in diameter) might induce terminal damage to live CMs and so are not suitable either (6, 7). Although successful isolation of viable single CMs using large-particle FACS with a nozzle of 500 μm in diameter has recently been reported (8), most published studies applying scRNA-seq in CMs have relied on either manual pick-up of CMs combined with plate-based methods (9) or the iCELL8 system, a large nozzle dispenser with a 5,184-nanowell plate and an imaging system to differentiate live cells from dead ones (10). An alternative solution is to isolate and sequence the single nuclei of CMs (11), which are smaller and can pass through FACS and droplet-based systems. Single-nucleus RNA sequencing (snRNA-seq), although is applicable in archived frozen specimens and can minimize the alteration of gene expression that may be caused by dissociation (12), should be used and its results interpreted with awareness of how it differs from scRNA-seq. A comparison of snRNA-seq and scRNA-seq showed that they both demonstrated similar cell clusters in the process of induced pluripotent stem cell (iPSC) differentiation to CMs. However, scRNA-seq captures more genes than snRNA-seq, represented by mitochondrial and ribosomal genes, while snRNA-seq detects more non-coding RNA and more reads that are mapped to intronic regions (11, 13). Importantly, the multinucleation and polyploidy of CMs also contribute to the divergence of snRNA-seq from scRNA-seq when applied to CMs, thus should be taken into consideration (14).

Besides the advances made in single-cell transcriptomics, its integration with single-cell epigenomics, namely, scATAC-seq, provides more information on both cell clustering and interactions between gene expression and open chromatin states (15, 16). Furthermore, spatial transcriptomics (i.e., technologies that recover transcriptomic information from tissue while preserving its spatial localization) is undergoing exponential development. Spatial transcriptomics uses either multiplexed RNA imaging [e.g., sequential fluorescence *in situ* hybridization (seqFISH)] or spatial barcoding (e.g., Visium, slide-seq) (5). Because the structure of the heart is complex and its tissues are highly heterogeneous, the addition of spatial information substantially improves our understanding of tissue architectures. It is also better able to uncover intercellular communications, thereby boosting the exploration of the underlying molecular pathways in the developing, normal, and diseased heart (5, 17).

In the following sections of this review, we focus on recent cardiovascular research that has applied single-cell omics technologies.

## Development of The Heart

Early scRNA-seq studies of the heart mainly focused on its development because fetal and neonatal cardiac tissues are easier to digest and their cells, including CMs, are smaller and so can pass through various single-cell platforms. Several such studies are highlighted in Table 1.

**Table 1:** Summary of studies of heart development.

**References**	**Species**	**Developmental stage**	**Target tissues/cells**	**Modalities**	**Major findings**
Chan et al. (18)	Mouse	Mouse ESC-derived embryoid bodies day 4	Dissociated cells from embryoid bodies	scRNA-seq	*Mesp*1^+^ mesoderm cells are highly heterogeneous
Lescroart et al. (19)	Mouse	Embryonic day 6.25 and 7.5	*Mesp*1^+^ or *Mesp1* KO CPCs	scRNA-seq	Mesp1 was essential for cell exit from the pluripotent state and the induction of the cardiac gene expression program
DeLaughter et al. (20)	Mouse	Embryonic day 9.5, 11.5, 14.5, and 18.5; postnatal day 0, 3, and 21	Microdissected embryonic heart tissues of each chamber	scRNA-seq	Single cells were classified into CMs, ECs, and fibroblast-enriched cells. Markers of temporal- and chamber-specific developmental programs were identified. *Nkx2-5* haploinsufficiency delayed developmental programs in both CMs and ECs
Li et al. (21)	Mouse	Embryonic day 8.5, 9.5, and 10.5	Microdissected embryonic heart tissues of each chamber	scRNA-seq	Using a random forest algorithm, the origins of single CMs were successfully reconstructed. Loss of *Nks2-5* led to failed differentiation into ventricular CMs
Jia et al. (15)	Mouse	Embryonic day 7.5, 8.5, and 9.5	*Isl*1^+^ or *Nkx*2−5^+^ CPCs	scRNA-seq and scATAC-seq	*Isl*1^+^ CPCs passed through an attractor state before segregating into different developmental branches. Continued expression of *Nkx2-5* established a unidirectional cardiomyocyte fate for CPCs
Xiong et al. (22)	Mouse	Embryonic day 7.75, 8.25, and 9.75	*Isl*1^+^ or *Nkx*2−5^+^ CPCs	scRNA-seq, CHIP-seq	CPCs in first and second heart fields exhibited different differentiation kinetics toward CMs. FHF cardiomyocytes guided the migration of SHF cells through the MIF-CXCR2/CXCR4 chemotaxis
Ivanovitch et al. (23)	Mouse	Embryonic day 6.9 (mid to late streak), day 7.1 (no-bud to early-bud), and day 7.3 (early headfold),	Tamoxifen treated *T^*nEGFP*−*CreERT*2/+^;R26R^*tdTomato*/*tdTomato*^* embryos	scRNA-seq	scRNA-seq demonstrated that the primitive streak cells contributing to the ventricles had a distinct molecular signature from those forming the outflow tract and atria, suggesting that cardiac progenitors were prepatterned in the primitive streak before migration.
de Soysa et al. (24)	Mouse	Embryonic day 7.75, 8.25, and 9.25	CPCs from control and *Hand2*-null embryos	scRNA-seq	*Hand2* was a specifier of outflow tract cells. *Hand2*-null mouse embryos failed to develop the right ventricle, which was due to failure of outflow tract myocardium specification
Xiao et al. (25)	Mouse	Embryonic day 13.5 and 14.5	Cardiac tissue from control or *Lats1/2* conditional (epicardium) KO mice	scRNA-seq	*Lats1/2* in EPDCs were essential for their differentiation into cardiac fibroblasts and for coronary vessel remodeling
Quijada et al. (26)	Mouse	Embryonic day 12.5 and 16.5; embryonic day 14.5	EPDCs from control at embryonic day 12.5 and 16.5; from conditional KO of myocardin-related transcription factors in the epicardium, embryonic day 14.5	scRNA-seq	*Slit*2^+^ EPDCs emerged following EMT. Genetic disruption of EMT altered the expression of vascular guidance cues such as *Slit2* and disturbed EC maturation and localization in the coronary vasculature
Sereti et al. (27)	Mouse	Embryonic day 9.5 and 12.5 and postnatal day 1	αMHC^+^ cardiomyocytes	scRNA-seq	Expression of cell cycle genes decreased to a minimal level postnatally, along with CM maturation
Su et al. (28)	Mouse	Embryonic day 12.5 and 14.5	Sinus venosus-derived ECs from control at embryonic day 12.5; from control and *Nr2f2* overexpression hearts at embryonic day 14.5	scRNA-seq	Vein cells underwent an early cell fate switch to pre-artery ECs. NR2F2 inhibited the pre-artery population *via* the induction of cell cycle genes
Li et al. (29)	Mouse	Embryonic day 10.5	Cardiac cells from dissected heart chambers and *Isl*1^+^ cardiac cells	scRNA-seq	CMs in G2/M phase downregulated sarcomeric and cytoskeletal markers
Wunnemann et al. (30)	Mouse	Embryonic day 14.5	Wild-type and *Adamts19*-null heart cells	scRNA-seq	*Adamts19* was a marker gene of valvular interstitial cells. Gene regulatory network analysis positioned *Adamts19* in a network downstream of the WNT signaling pathway
Hulin et al. (31)	Mouse	Postnatal day 7 and 30	Cells from aortic and mitral valve leaflets	scRNA-seq	Subpopulations of endothelial and immune cells were comparable between the two time points, whereas interstitial cells were more diverse at postnatal day 30, expressing complement factors, ECM proteins, and osteogenic genes
Wang et al. (32)	Mouse	Postnatal day 1, 4, 7, 14 and 56	CMs and non-CMs from left ventricles	scRNA-seq	Genes involved in ECM organization, BMP signaling pathway and CM differentiation were enriched in mature fibroblasts when compared with immature ones. Maturation of CMs were promoted when co-cultured with adult fibroblasts, but not with neonatal fibroblast.
Goodyer et al. (33)	Mouse	Embryonic day 16.5	Cells from three zones of microdissected hearts: sinoatrial node region, atrioventricular node/His region, and bundle branch/Purkinje fiber region	scRNA-seq	The study revealed clusters of sinoatrial node cells, atrioventricular node/His cells, and Purkinje fiber and transitional Purkinje fiber cells, as well as their cell subtypes, and thus delineated the first transcriptional landscape of the developing cardiac conduction system
Suryawanshi et al. (34)	Human	19–22 weeks gestation	Cells from healthy or autoimmune-associated CHB fetal hearts	scRNA-seq	Fetal heart with autoimmune-associated CHB exhibited varying degrees of increased interferon responses in all cell types. Matrisome transcripts were highly enriched in the fibroblasts and smooth muscle cells in the CHB sample
Sahara et al. (35)	Human	Human ESC-derived cardiac lineages day 3, 6, and 15; human embryonic/fetal hearts 4.5–10 weeks gestation	Human ESC-derived cardiac lineages; human embryonic/fetal cardiac cells	scRNA-seq	Cono-ventricular progenitors were marked by *LGR5*, which is human-specific. *LGR*5^+^ progenitors promoted cardiogenesis *via* expansion of committed cardiac intermediates
Cui et al. (36)	Human	5–25 weeks gestation	Anatomically informed cardiac cells from human embryos	scRNA-seq	The study identified major cell types and demonstrated the similarities in and differences between humans and mice
Lahm et al. (37)	Human/mouse	4.5–10 weeks gestation and adults	Human embryonic/fetal cardiac cells; adult human atrial and ventricular cells	scRNA-seq	The four detected genes—*MACROD2, GOSR2, WNT3*, and *MSX1*—were highly expressed throughout development, and *MACROD2* remained expressed in adult heart
Asp et al. (38)	Human	4.5–5, 6.5, and 9 weeks post-conception	Human embryonic/fetal cardiac cells	scRNA-seq, spatial barcoding, and *in situ* sequencing	The study generated the first spatiotemporal cell atlas for the developing human heart
Tyser et al. (39)	Mouse	Embryonic day 7.75–8.25 (divided into six stages)	Microdissected anterior cardiac region	scRNA-seq, multiplexed RNA imaging	A previously unknown CPC pool marked by *Mab21l2* was identified, which contributed to not only CMs, but also the epicardium
Mantri et al. (40)	Chicken	Embryonic day 4, 7, 10, and 14 (corresponding to HH21–24, HH30–31, HH35–36, and ~HH40)	Enzymatically digested cardiac ventricular tissues	scRNA-seq, spatial barcoding, and multiplexed RNA imaging	Spatially restricted genes during development were identified. The study also discovered a *TMSB4X*-expressing subpopulation that overlapped coronary vascular cells throughout coronary development

*CHB, congenital heart block; ChIP-seq, chromatin immunoprecipitation with parallel sequencing; CM, cardiomyocyte; CPC, cardiac progenitor cell; EC, endothelial cell; EMT, epithelial-to-mesenchymal transition; ESC, embryonic stem cell; FHF, first heart field; HH, Hamburger-Hamilton; KO, knockout; scATAC-seq, single-cell assay for transposase-accessible chromatin sequencing; scRNA-seq, single-cell RNA sequencing; SHF, second heart field*.

It had been unclear whether *Mesp*1^+^ mesoderm cells were intrinsically heterogeneous or were homogeneous but capable of multiple lineage decisions. Taking advantage of scRNA-seq, Chan et al. unraveled the heterogeneity of *Mesp*1^+^ mesoderm cells from mouse embryonic stem cell (ESC)-derived embryoid bodies (18). Lescroart et al., by examining *Mesp*1^+^ cardiac progenitor cells (CPCs) as well as *Mesp1*-expressing cells in the *Mesp1* knockout context, found that *Mesp1* was essential for exit from the pluripotent state and for induction of the cardiac gene expression program (19).

Efforts to establish a transcriptomic atlas of the developing heart have been undertaken by multiple teams. By investigating >1,200 cells from murine heart chambers of different developmental stages, DeLaughter et al. not only successfully clustered CMs, endothelial cells (ECs), and fibroblast-enriched cells, but also identified markers of temporal- and chamber-specific developmental programs (e.g., genes involved in cell cycle at embryonic day 9.5–11.5 and genes involved in cellular growth and CM differentiation between embryonic day 14.5 and postnatal day 3) (20). Li et al. used a similar approach together with a random forest algorithm to reconstruct the spatial origins of single CMs (21). Both studies demonstrated that *Nkx2-5* deficiency led to failure of CM maturation (20, 21). Jia et al. integrated scRNA-seq and scATAC-seq in *Nkx*2−5^+^ and *Isl*1^+^ CPCs, enabling developmental trajectories to be reconstructed and revealing that *Isl*1^+^ CPCs passed through an attractor state before segregating into different developmental branches, whereas continued expression of *Nkx2-5* established a unidirectional CM fate for CPCs (15). A similar analysis of *Nkx*2−5^+^ and *Isl*1^+^ CPCs found interactions between first and second heart field CPCs, with second heart field CPCs attracted to the first heart field-populated heart tube region through MIF (macrophage migration inhibitory factor)-CXCR2/CXCR4 chemotaxis, contributing to the growth of the outflow tract and right ventricle (22). Furthermore, using genetic lineage tracing and live imaging, Ivanovitch et al. discovered the temporal order in which different cardiac lineages arose within the primitive streak: the progenitors of the left ventricle at the mid-streak stage, those of the right ventricle at the late-streak stage, and those of the outflow tract and atria at the no-bud to early-bud stage. More importantly, scRNA-seq demonstrated distinct molecular signatures of these subpopulations of primitive streak cells, indicating their prepatterned fate in the primitive streak before migration (23). Using a Boolean network-based lineage-specifier prediction method in the downstream analysis of scRNA-seq of mouse embryos as an alternative approach, de Soysa et al. identified *Hand2* as a specifier of outflow tract cells. *Hand2*-null mouse embryos failed to specify outflow tract myocardium, whereas the right ventricle myocardium was specified but not properly differentiated and migrated, which was associated with disrupted anterior–posterior patterning of CPCs (24).

With a focus on epicardium-derived cells (EPDCs), Xiao et al. demonstrated that epicardium conditional knockout of *Lats1/2*, encoding LATS1 and LATS2 that are negative regulators in the Hippo signaling pathway, caused arrested differentiation into fibroblasts and defective coronary vasculature remodeling (25). Quijada et al. also investigated epicardial contributions to the growing coronary plexus by applying scRNA-seq to EPDCs and uncovered a *Slit*2^+^ subpopulation that emerged following epithelial-to-mesenchymal transition (EMT). Genetic disruption of EMT altered the expression of vascular guidance cues such as *Slit2* and disturbed EC maturation and localization in the coronary vasculature (26). Su et al. analyzed sinus venosus-derived ECs, discovered that the vein cells underwent an early cell fate switch to pre-artery ECs, and identified the transcription factor NR2F2 as a vein specifier, whose overexpression prevented differentiation to the pre-artery population *via* the induction of cell cycle genes (28). By performing a genome-wide association study (GWAS) in humans, Wunnemann et al. detected mutations in *ADAMTS19* that are responsible for valvular diseases. They then applied scRNA-seq to wild-type and *Adamts19*-null murine heart at embryonic day 14.5 and identified *Admats19* as a marker of valve interstitial cells. Gene regulatory network analysis helped to identify the Wnt-Adamts19-Klf2 axis as being essential for valve development (30). Another study of murine postnatal valve leaflets demonstrated that, whereas subpopulations of ECs and immune cells were comparable between day 7 and 30, interstitial cells were more diverse at postnatal day 30, expressing complement factors, extracellular matrix proteins, and osteogenic genes (31). Wang et al. analyzed the dynamic change of subtypes of non-CMs along with postnatal CM maturation in murine hearts. scRNA-seq of CMs and non-CMs of murine hearts at postnatal day 1, 4, 7, 14, and 56 showed that fibroblasts increasingly expressed maturation promoting genes such as *Dcn*. Co-culture experiments demonstrated that maturation of CMs was promoted when co-cultured with adult fibroblasts, but not with neonatal fibroblasts. Their results indicated fibroblasts subtype switching as a crucial microenvironmental event regulating postnatal CM maturation (32).

Development of the conduction system has also attracted researchers' interest. Goodyer et al. performed scRNA-seq on cells from murine embryonic heart regions including the conduction system. The study revealed clusters of sinoatrial node cells, atrioventricular node/His cells, and Purkinje fiber and transitional Purkinje fiber cells, thus delineated the first transcriptional landscape of the developing cardiac conduction system (33). Suryawanshi et al. investigated single-cell transcriptomes from healthy human fetal hearts and a fetal heart with autoimmune-associated congenital heart block and showed, through a comparison with normal fetal hearts, that the fetal heart with autoimmune-associated congenital heart block exhibited increased interferon responses of varying degrees in all cell types. Additionally, matrisome transcripts were highly enriched in the fibroblasts and smooth muscle cells in the congenital heart block sample compared with healthy hearts, indicating their contribution to fibrosis (34).

Other studies of human heart development, although rare, have also been conducted to unravel the human-specific features. Cui et al. identified cell types from human fetal hearts and compared their gene expression profiles with those of mice (36). A study of human ESC-derived cardiac derivates and human embryonic/fetal hearts revealed a human-specific cono-ventricular progenitor population marked by *LGR5*. LGR5 (leucine rich repeat containing G protein-coupled receptor 5) is a receptor for R-spondins, which is involved in the WNT signaling pathway. *LGR*5^+^ progenitors were shown to promote cardiogenesis *via* expansion of committed cardiac intermediates (35). Lahm et al. identified four congenital heart disease-associated genes—*MACROD2, GOSR2, WNT3*, and *MSX1*—by GWAS and explored their expression in embryonic and adult human heart by scRNA-seq. They found that *MACROD2, GOSR2, WNT3*, and *MSX1* were highly expressed throughout development and that *MACROD2* expression persisted in adult heart (37).

More recent research implemented multiple advanced technologies along with scRNA-seq. Asp et al. generated the first spatiotemporal cell atlas for human developing heart by integrating scRNA-seq, spatial barcoding, and multiplexed RNA imaging (38). Tyser et al., by combining scRNA-seq with genetic lineage labeling and abundant imaging modalities including multiplexed RNA *in situ* hybridization in murine embryonic hearts, identified and located the CPC subpopulations. *Via* this spatial transcriptomic approach, they unraveled a previously unknown progenitor subpopulation marked by *Mab21l2* that located at the rostral border of the cardiac crescent and contributed to both CMs and epicardium. Mab21l2 (Mab-21 like 2) was previously known to be required for neural and eye development, was here implied to represent the earliest progenitors of the epicardium (39). By integrating scRNA-seq and spatial transcriptomics in developing chicken hearts, Mantri et al. identified spatially restricted genes in cardiac tissue during development. They found that *TBX5* was enriched in the left ventricle compared with the right but decreased from embryonic day 7 to 14 and that *CHGB* expression was restricted to the right ventricle from day 7 onward. In addition, they discovered a *TMSB4X*-expressing subpopulation that overlapped with coronary vascular cells throughout coronary development. TMSB4 (thymosin β4) participates in regulating actin polymerization, thus is involved in cell proliferation, migration, and differentiation. Its role in heart development had long been under significant debate, given the paradoxical results of several previous *Tmsbx* knockout and knockdown experiments in mouse models, and was provided deeper insights into by this study (40). Undoubtedly, the latest technological advances, represented by spatial transcriptomics, are furthering our understanding of the molecular mechanisms of cardiac development.

## Homeostasis of The Adult Heart

Single-cell omics approaches have also been implemented in studies of adult mice and humans in homeostasis (Table 2), with more studies focusing on non-CMs than on CMs. Yekelchyk et al. performed scRNA-seq on murine CMs using the iCELL8 platform, finding that gene expression was homogeneous between mono- and multi-nucleated CMs in heathy ventricles (41). By performing scRNA-seq on non-CMs of murine heart, Skelly et al. detected major cell types and transcriptional heterogeneity, revealed communications between cell types, and showed cell type-specific sexual dimorphism of cardiac gene expression, such as male upregulation of *Irf8* (interferon regulatory factor 8) and female upregulation of *Tsc22d3* (TSC22 domain family member 3, a transcription factor implicated in anti-inflammatory functions) in macrophages. Their results provided molecular cues for the sexual difference of cardiac responses to the environmental insults (42). Chakarov et al. identified two distinct interstitial macrophage populations occupying different niches, which were conserved across lung, heart, fat, and dermis tissues: Lyve1^low^MHCII^high^CX3CR1^high^ and Lyve1^high^MHCII^low^CX3CR1^low^ monocyte-derived interstitial macrophages. They also demonstrated that depletion of Lyve1^high^MHCII^low^CX3CR1^low^ interstitial macrophages exacerbated lung and heart fibrosis (43), which was highly consistent with the results obtained using a mouse MI model (55). Macrophages also facilitate conduction in the atrioventricular node. Genes involved in cardiac conduction are enriched in macrophages from the atrioventricular node when compared with those from other tissues. Atrioventricular node macrophages highly express *Cx43*, contributing to the gap junctions that link them to neighboring CMs (44). In regard to the conduction system, quantitative proteomics of the murine sinus node demonstrated a remarkable abundance of ion channels responsible for the pacemaking process (e.g., HCN4), which were indicated by snRNA-seq to be predominantly expressed by sinus node myocytes (45). Liang et al. then verified the existence of a cell cluster in the sinus node marked by *Hcn1* and *Hcn4*, where *Vsnl1* was also highly expressed. VSNL1 (visinin-like 1) is a member of visinin/recoverin subfamily of neuronal calcium sensor proteins, and is involved in calcium signaling pathways. Adeno-associated virus-mediated knockdown of *Vsnl1* in mice resulted in a reduced heart rate and decreased expression of *Hcn4, Cacna1d, Cacna1i*, and *Serca2a*, but not that of *Ryr2* or of the genes involved in potassium channels (46).

**Table 2:** Summary of studies of homeostasis of the adult heart.

**References**	**Species**	**Target tissues/cells**	**Modalities**	**Major findings**
Yekelchyk et al. (41)	Mouse	CMs from both healthy and hypertrophic ventricles	scRNA-seq	Gene expression was homogeneous between mono- and multi-nucleated CMs in homeostasis. Heterogeneity was introduced by TAC
Skelly et al. (42)	Mouse	Non-CMs from the heart	scRNA-seq	Major cell types and transcriptional heterogeneity were detected, as well as communications among cell types. The study also showed cell type-specific sexual dimorphism of cardiac gene expression
Chakarov et al. (43)	Mouse	Lung interstitial macrophages (validated in the heart, fat, and skin by flow cytometry)	scRNA-seq	Two distinct IM populations conserved across the lung, heart, fat, and dermis tissues were identified. Depletion of Lyve1^high^MHCII^low^CX3CR1^low^ IMs exacerbated lung and heart fibrosis
Hulsmans et al. (44)	Mouse	Macrophages in the AV nodes	scRNA-seq	Genes involved in cardiac conduction were enriched in macrophages from the AV node compared with those from other tissues. AV node macrophages highly expressed *Cx43*, forming the gap junctions linking them to neighboring CMs
Linscheid et al. (45)	Mouse	Cells from sinus node tissue and the adjacent atrium	snRNA-seq, bulk proteomics	Quantitative proteomics of murine sinus node demonstrated significant abundancy of ion channels responsible for the pacemaking process (e.g., HCN4), which were predominantly expressed by sinus node myocytes, as revealed by snRNA-seq
Liang et al. (46)	Mouse, rabbit, monkey	Cells from microdissected sinus node tissues and those from atrial and ventricular CMs	scRNA-seq	A cell cluster expressing *Hcn1, Hcn4*, and *Vsnl1* was found. Knockdown of *Vsnl1* in mice resulted in reduced heart rate and decreased *Hcn4, Cacna1d, Cacna1i*, and *Serca2a* expression, but not that of *Ryr2* or those involved in potassium channels
Paik et al. (47)	Mouse	scRNA-seq data from tissue-specific ECs from the Tabula Muris	scRNA-seq	Cardiac ECs could be classified as a separate cluster but had considerable transcriptomic overlap with ECs from other tissues. Markers of tissue-specific ECs identified in mice were also enriched in their corresponding human tissue-specific ECs
Yucel et al. (48)	Mouse	ECs and non-ECs from murine heart	Bulk RNA-seq, snRNA-seq, bulk ATAC-seq, multiplexed RNA imaging	Cardiac ECs actively expressed cardiac myofibril genes such as *Tnnt2* and *Myh6* and had open chromatin at cardiac myofibril gene promoters
Hu et al. (49)	Human	Cells from human aorta, pulmonary artery, and coronary arteries collected from patients undergoing heart transplantation	scRNA-seq	Artery-specific cell subpopulations with distinct transcriptional profiles were identified in VSMCs and fibroblasts, but not in ECs. Intercellular communication between macrophages and ECs was predicted to increase in atherosclerosis
Wolfien et al. (50)	Mouse	Nuclei isolated from entire murine hearts	snRNA-seq	Distinct cell clusters were identified, including immune cells and cells of neuronal origin. RNA velocity enabled interrogation of transcriptional kinetics
Vidal et al. (51)	Mouse	Nuclei isolated from entire hearts of 12-week-old and 18-month-old mice	snRNA-seq	Angiogenesis-related extracellular protein-encoding genes including *Serpine1* and *Serpine2* were enriched in fibroblasts derived from aged hearts, indicating an affected paracrine crosstalk between fibroblasts and ECs during aging. Conditioned medium derived from aged fibroblasts had higher levels of serpins and showed a reduced angiogenic property, which was mediated by an impairment of EC function
Tucker et al. (52)	Human	Tissue samples taken from the lateral aspect of the four cardiac chambers from potential transplant donors	snRNA-seq	CMs were the most heterogeneous of various cell types. When combined with GWAS data, genes at the loci associated with heart rhythm were enriched in CMs, whereas those associated with CAD were enriched in pericytes
Litvinukova et al. (53)	Human	Full-thickness myocardial biopsies from the left and right atria, left and right ventricles, and interventricular septum and apex from deceased transplant organ donors	scRNA-seq, snRNA-seq, and multiplexed RNA imaging	CMs, pericytes, and fibroblasts were the most heterogeneous. Predicted intercellular communications among CMs, fibroblasts, and immune cells differed between atria and ventricles
Wang et al. (54)	Mouse	Murine heart non-myocytes	scRNA-seq, scATAC-seq	Differential accessibilities of the *cis*-regulatory elements among different subpopulations in each cell type that potentially regulate the expression of marker genes

*ATAC-seq, assay for transposase-accessible chromatin sequencing; AV, atrioventricular; CAD, coronary artery disease; CM, cardiomyocyte; GWAS, genome-wide association study; IM, interstitial macrophage; EC, endothelial cell; scATAC-seq, single-cell assay for transposase-accessible chromatin sequencing; scRNA-seq, single-cell RNA sequencing; snRNA-seq, single-nucleus RNA sequencing; TAC, transverse aorta constriction; VSMC, vascular smooth muscle cell*.

Cardiac ECs are another research interest. Paik et al. re-explored the scRNA-seq data from tissue-specific ECs in the Tabula Muris (4) and demonstrated that cardiac ECs could be classified as a separate cluster, even though they had considerable transcriptomic overlap with ECs from other tissues (47). Yucel et al. unexpectedly observed that adult murine cardiac ECs actively expressed cardiac myofibril genes such as *Tnnt2* and *Myh6*, which was consistent with the analytical results of multiple datasets; this result was validated by RNA *in situ* hybridization. They also showed that cardiac ECs had open chromatins at cardiac myofibril gene promoters, suggesting that the results were not due to technical contamination or paracrine transfer of mRNA. Interestingly, the accessibility of cardiac myofibril genes was no longer maintained in cardiac ECs upon culture. Although the significance of the expression of cardiac myofibril genes in cardiac ECs is still unclear, the authors hypothesized that those genes played a role in maintaining cardiac EC maturity and specificity, which needs further investigation (48). Hu et al. used scRNA-seq to analyze cells from human aorta, pulmonary artery, and coronary arteries collected from patients who underwent heart transplantation. Artery-specific cell subpopulations with distinct transcriptional profiles were identified in vascular smooth muscle cells and fibroblasts, but not in ECs (49).

Recently, efforts have focused on depicting the full picture of the cell atlas of the whole heart. snRNA-seq of murine hearts successfully clustered distinct cell subpopulations, including immune cells, cells of neuronal origin, and cardiac glial cells (50). With snRNA-seq, Vidal et al. investigated the transcriptomes of murine hearts of 12-week-old and 18-month-old, and found upregulated angiogenesis-related extracellular protein-encoding genes in aged hearts, indicating an affected paracrine crosstalk between fibroblasts and ECs during aging. *In vitro* experiments confirmed that conditioned medium derived from aged fibroblasts showed a reduced angiogenic property, which was mediated by an impairment of EC function (51). In humans, samples of myocardium from cardiac chambers, rather than of the whole heart, have been studied. By applying snRNA-seq to biopsied human cardiac chambers, Tucker et al. showed that CMs were the most spatially heterogeneous of the distinct cell types. Furthermore, by combining the snRNA-seq results with GWAS data for cardiometabolic traits, they revealed that genes at the loci associated with heart rhythm were enriched in CMs, whereas those at the loci associated with coronary artery disease were enriched in pericytes (52). Litvinukova et al. investigated full-thickness biopsy samples from deceased transplant donors and were able to provide more in-depth information, predicting different intercellular communications among immune cells, CMs, and fibroblasts between ventricles and atria (53). Wang et al. integrated scRNA-seq and scATAC-seq for non-CMs in murine hearts and revealed the heterogeneity of non-CM populations and their subpopulations at transcriptomic and epigenomic levels. Cardiac fibroblasts were classified into three distinct functional states related to response to stimuli (marked by *Hsd11b1*; 11β-hydroxysteroid dehydrogenase type 1), the cytoskeleton (marked by *Gfpt2*; glutaminefructose-6-phosphate transaminase 2, involved in the hexosamine biosynthesis pathway), and immune regulation (marked by *C1qa*), respectively. Importantly, they also identified differential accessibilities of the *cis*-regulatory elements among different subpopulations of each cell type that potentially regulate the expression of marker genes (54). The success of these studies indicates that single-cell omics represents an indispensable toolbox for broadening our insights into the homeostasis of the adult heart, particularly the heterogeneity of cells and their functions, as well as intercellular communications.

## Myocardial Infarction/Ischemia-Reperfusion Injury

In addition to development and adult homeostasis, single-cell omics approaches have played a crucial role in research into diseased hearts (Table 3). MI, as a consequence of atherosclerotic disease, may have a marked effect on cardiac function and quality of life. The toolbox from single-cell omics technologies has enabled detailed investigation of not only the cells involved in the process of plaque formation and rupture, but also the ischemic myocardium that is susceptible to remodeling (71).

**Table 3:** Summary of studies of myocardial infarction or ischemia-reperfusion injury.

**References**	**Species**	**Ischemia model**	**Target tissues/cells**	**Modalities**	**Major findings**
King et al. (56)	Mouse	Permanent ligation of left coronary artery	Leukocytes isolated from wild-type and *Irf3*-null heart at day 4 post-MI or sham	scRNA-seq	MI induced an IRF3-dependent type I interferon response in a distinct subpopulation of cardiac macrophages. Interruption of IRF3-dependent signaling decreased the cardiac expression of inflammatory cytokines and chemokines and improved cardiac function and survival
Vafadarnejad et al. (57)	Mouse	Permanent ligation of LAD	Neutrophils isolated from infarcted hearts and blood at day 1, 3, and 5 post-MI or sham	scRNA-seq, CITE-seq	Post-MI cardiac neutrophils were temporally heterogeneous. Infiltrating neutrophils were demonstrated to locally acquire a SiglecF^high^ state at day 3 onward after MI, in which transcripts associated with neutrophil aging and activation were also enriched
Xia et al. (58)	Mouse	Permanent ligation of LAD; 60-min ligation of LAD	Regulatory and conventional T cells from heart, spleen, non-draining LNs, and mediastinal LNs at day 7 post-MI or sham	Bulk RNA-seq, scRNA-seq, scTCR-seq	Tregs were recruited to the injured myocardium after MI or I/R injury from the circulating Treg pool. A considerable fraction of heart Tregs was clonally expanded. ECM-associated genes, including Sparc, were enriched in heart Tregs. Heart Tregs led to increased collagen content and prevented rupture, with Sparc playing a critical role in this process
Heinrichs et al. (59)	Mouse	Permanent ligation of LAD	B cells from heart and mediastinal LNs at day 5 post-MI or sham	scRNA-seq, scBCR-seq	Cardiac B cells accumulated rapidly after MI *via* the CXCL13-CXCR5 axis. These cells were largely polyclonal and contributed to local TGFβ production. CXCR5-deficiency reduced B cell infiltration and local *Tgfb1* expression but did not change post-MI contractile function or myocardial morphology
Gladka et al. (60)	Mouse	75-min ligation of left coronary artery	Cells from the infarct and border zone region from infarcted heart at day 3 post-MI or sham	scRNA-seq	A subcluster of fibroblasts was revealed to be specific to injured hearts, highly expressing *Ckap4*, along with well-known myofibroblast markers such as *Postn* and *Cthrc1*. *In vitro* inhibition of *Ckap4* resulted in increased expression of myofibroblasts after TGFβ induction
Molenaar et al. (61)	Mouse	60-min ligation of LAD	Cells from non-infarct regions from the heart at day 1, 3, and 14 post-injury or sham	scRNA-seq	*Mfge8, Calr* and *B2m* were upregulated in CMs at day 1 post-injury, with their cognate receptors expressed in other cell types (dominantly in fibroblasts). B2M induced the expression of myofibroblast markers in fibroblasts *in vitro*
Farbehi et al. (62)	Mouse	Permanent ligation of LAD	All non-CMs and enriched (*Pdgfra*-GFP^+^) fibroblast lineage cells from ventricles at day 3 and 7 post-MI or sham	scRNA-seq	A novel fibroblast subpopulation expressing *Wif1* was discovered in both sham and post-MI hearts, which presented an anti-WNT/CTGF/TGFβ signature, and interacted with ECs. IF revealed WIF1^+^ fibroblasts in the border zone at post-MI day 3, but not in sham or post-MI day 1 or 7 hearts, indicating the post-transcription and injury-dependent regulation of WIF expression, and its function of inhibiting fibrosis and angiogenesis
Kretzschmar et al. (63)	Mouse	Permanent ligation of LAD; 60-min ligation of LAD	Cells from neonatal heart or adult heart 14 days after MI, I/R, or sham	scRNA-seq	By investigating murine heart 14 days after MI with genetic lineage tracing using *Ki67* knockin models, no cycling CMs were found in infarcted hearts. A subpopulation of proliferative fibroblasts expressing *Fstl1* was identified, which resembled neonatal cardiac fibroblasts. Conditional KO of *Fstl1* in fibroblasts resulted in more rupture
Forte et al. (64)	Mouse	Permanent ligation of LAD	Cardiac interstitial cells at post-MI day 1, 3, 5, 7, 14, and 28 or sham	scRNA-seq	Epicardial-derived injury-response fibroblasts arose immediately after MI and were replaced by myofibroblasts, followed by matrifibrocytes and late-response fibroblasts. Different strains of mice exhibited different post-MI dynamics of fibroblasts, which was related to cardiac rupture
Yokota et al. (65)	Mouse	Permanent ligation of LAD	Non-CMs from heart at post-MI day 7 in wild-type and *Col5a* conditional KO (in fibroblasts) mice	scRNA-seq	Deficiency in type V collagen resulted in a paradoxical increase in post-MI scar tissue with altered mechanical properties of scars and myofibroblast induction
Li et al. (66)	Mouse	Permanent ligation of LAD	Cardiac ECs from post-MI day 7 or sham heart in EC-specific multispectral lineage tracing mice	scRNA-seq	Clonal proliferation of resident ECs occurred in the infarct border zone. Subpopulations of ECs expressing *Plvap* increased after MI. *In vitro* inhibition of Plvap reduced EC proliferation
Tombor et al. (67)	Mouse	Permanent ligation of LAD	Cardiac non-CMs from post-MI day 1, 3, 5, 7, 14, and 28 or sham	scRNA-seq	ECs underwent transient mesenchymal transition on days 3–7 after MI
Gladka et al. (68)	Mouse	Permanent ligation of LAD	Cells from the infarct and border zone region from infarcted heart at day 3 post-MI or sham	scRNA-seq	scRNA-seq revealed upregulated *Zeb2* expression in injured CMs, which facilitated release of TMSB4 and PTMA. Overexpression of *Zeb2, Tmsb4x*, and *Ptma* in CMs enhanced EC migration and proliferation *in vitro*
Kuppe et al. (69)	Human	-	Heart tissues and isolated cells from patients with acute/chronic MI or non-transplanted donor	snRNA-seq, snATAC-seq, spatial barcoding	Spatially distinct CM, fibroblast, and EC subclusters were identified. Trajectory analysis from snRNA-seq and snATAC-seq data revealed an increase in both the expression and transcription factor motif accessibility of *RUNX1*, along with myofibroblast differentiation
Zhang et al. (70)	Mouse	Permanent ligation of LAD	Isolated nuclei from ventricles at post MI day 5 or sham, from triple-transgenic linage tracing mice (*αMHC-MerCreMer*; *RFP^*fl*^/GFP*; *Myh6-H2BBFP6xHis)*	snRNA-seq	snRNA-seq detected an increased BFP^−^ proportion in post-MI CMs, within which *Mki67, Runx1* and cell cycle genes were expressed in a higher level, pathways for cardiac contraction and rhythm were downregulated, and survival/proliferation-related pathways and pathways for extracellular matrix receptor interaction were upregulated. This population was thought to represent the dedifferentiated CMs post-MI

*ATAC-seq, assay for transposase-accessible chromatin sequencing; CITE-seq, cellular indexing of transcriptomes and epitopes by sequencing; CM, cardiomyocyte; EC, endothelial cell; ECM, extracellular matrix; IF, immunofluorescence; I/R, ischemia/reperfusion; KO, knockout; LAD, left anterior descending artery; LN, lymph node; MI, myocardial infarction; RNA-seq, RNA sequencing; scBCR-seq, single-cell B cell receptor sequencing; scRNA-seq, single-cell RNA sequencing; scTCR-seq, single-cell T cell receptor sequencing; Treg, regulatory T cell*.

MI has long been known to involve both pro- and anti-inflammatory cascades. King et al. were the first to apply scRNA-seq to leukocytes isolated from infarcted and non-infarcted murine hearts, finding that MI induced an IRF3-dependent type I interferon response in a distinct subpopulation of cardiac macrophages and that interruption of IRF3-dependent signaling decreased the cardiac expression of inflammatory cytokines and chemokines and improved cardiac function (56). Cardiac-infiltrating neutrophils were demonstrated to locally acquire a SiglecF^high^ ICAM^high^CXCR4^high^ state from day 3 after MI, indicating local neutrophil activation and aging. Similar neutrophil subpopulation was also found in atherosclerotic aortas, suggesting their roles both in acute and chronic cardiovascular inflammation (57). Regulatory T cells (Tregs) accumulated in the injured myocardium after MI or ischemia-reperfusion injury. The majority of these heart Tregs were derived from the circulating Treg pool; a considerable fraction of them experienced clonal expansion and displayed a unique T-cell receptor repertoire. Importantly, extracellular matrix-associated genes, including *Sparc*, were enriched in heart Tregs compared with other Tregs. Further experiments revealed that heart Tregs contributed to increased collagen content and prevented rupture and that *Sparc* was critical in this process (58). After MI, cardiac B cells also accumulated rapidly *via* the CXCL13-CXCR5 axis but were largely polyclonal and contributed to local TGFβ production, indicating their involvement in fibroblast activation (59).

Fibroblasts are directly involved in post-infarct scar formation. Gladka et al. performed scRNA-seq on cells sorted by FACS with a 130-μm nozzle from infarcted and control murine hearts and were able to identify distinct clusters of CMs, fibroblasts, ECs, and macrophages. A subset of activated fibroblasts was revealed to be specific to injured hearts and to strongly express *Ckap4*. CKAP4 (cytoskeleton associated protein 4) is a transmembrane receptor whose function in cardiac fibroblasts had remained unknown. *In vitro* inhibition of *Ckap4* resulted in increased expression of genes related to activated fibroblasts after TGFβ induction, suggesting a modulating function for CKAP4 in fibroblast activation (60). Molenaar et al. focused on the ligands upregulated in CMs 1 day after ischemia, whose cognate receptors were expressed by another cell type, and detected *B2m* (β2 microglobulin) in CMs and its receptors in fibroblasts. *In vitro* experiments demonstrated that fibroblasts expressed myofibroblast markers when cultured with B2M (61). Farbehi et al. discovered a novel fibroblast subpopulation with upregulated *Wif1* expression, in sham hearts, which persisted after MI, had an anti-WNT/CTGF/TGFβ signature, and interacted with ECs. Immunofluorescence revealed WIF1^+^ fibroblasts in close proximity with ECs in the border zone at post-MI day 3, but not in sham or post-MI day 1 or 7 hearts, indicating the injury-dependent and post-transcriptional regulation of WIF expression, and its function of inhibiting fibrosis and angiogenesis in post-MI repair (62). By investigating the murine heart 14 days after MI, Kretzschmar et al. identified a subpopulation of proliferative fibroblasts resembling neonatal cardiac fibroblasts, which presented with upregulated *Fstl1* (follistatin-like 1, which had been known to be expressed in adult cardiac fibroblasts). Conditional knockout of *Fstl1* in fibroblasts induced less proliferative cells at the infarct and border zones and lead to more cardiac ruptures, indicating the importance of autocrine FSTL1 signaling in preventing rupture (63). Longitudinal scRNA-seq analysis of murine heart interstitial cells from post-MI day 1 to 28 uncovered a dynamic shift in the subpopulations. In particular, epicardial-derived injury-response fibroblasts arose immediately after MI and were replaced by myofibroblasts, followed by matrifibrocytes and late-response fibroblasts. Different strains of mice exhibited different post-MI dynamics of fibroblasts, which was related to the risk of cardiac rupture (64). Yokota et al. demonstrated that a deficiency in type V collagen resulted in a paradoxical increase of scar tissue, where mechanical properties of scars were altered, and myofibroblasts were induced (65).

ECs are involved in angiogenesis after MI and have thus been another target of single-cell studies. Li et al. discovered that clonal proliferation of resident ECs occurred in the infarct border zone, in which *Plvap* was enriched. PLVAP (plasmalemma vesicle associated protein) had been known as an EC-specific membrane protein involved in the formation of transendothelial channels and microvascular permeability. *In vitro* inhibition of *Plvap* reduced EC proliferation, indicating its involvement in neoangiogenesis (66). Li et al. also observed transient endothelial mesenchymal activation, but the extent to which was comparable between post-MI day 7 and sham. By contrast, Tombor et al. demonstrated that ECs underwent transient mesenchymal activation on days 1 to 7 after MI, which returned to homeostasis afterwards, indicating its involvement in facilitating regeneration of the vascular network *via* EC migration and clonal expansion (67). A crosstalk between injured CMs and ECs has also been identified: transcription factor *Zeb2* expression was upregulated in injured CMs, which facilitated the release of TMSB4 and PTMA (prothymosin α). Overexpression and knockdown experiments of *Zeb2, Tmsb4x*, and *Ptma* in iPSC-derived CMs clearly demonstrated their involvement in enhancing EC proliferation and migration (68).

Kuppe et al. integrated snRNA-seq, single-nucleus assay for transposase-accessible chromatin sequencing (snATAC-seq), and spatial transcriptomics in human heart tissues from patients with and without a history of MI. Trajectory analysis from snRNA-seq and snATAC-seq data revealed an increase in both the expression and the transcription factor motif accessibility of *RUNX1* in fibroblasts, as well as in their differentiation to myofibroblasts. *In vitro* experiments then validated that *RUNX1* amplified TGFβ signaling and thus mediated myofibroblast differentiation (69). Zhang et al. developed a triple-transgenic mouse model (α*MHC-MerCreMer*; *RFP*^*fl*^*/GFP*; *Myh6-H2BBFP6xHis*, tamoxifen treated) for fate mapping of CMs after MI. They discovered an increase in the rare GFP^+^BFP^low^ CMs in post-MI hearts compared to sham hearts that presented with a high BrdU^+^ incorporation rate, which were thought to represent dedifferentiated CMs. snRNA-seq also detected an increased BFP^−^ proportion in post-MI CMs, within which *Mki67, Runx1*, and cell cycle genes were expressed in a higher level. They further showed that pathways for cardiac contraction and rhythm were downregulated, and pathways for survival/proliferation and extracellular matrix receptor interaction were upregulated (70).

MI is not a local event but rather a spatiotemporally heterogeneous state of disease involving the entire heart. The conventional approach for increasing the spatial resolution in earlier studies was dissecting infarct hearts into ischemic zone, border zone, and remote zone. To this end, spatial transcriptomics adds a continuous spatial resolution to the transcriptomic information, which is being used in ongoing research into MI.

## Heart Failure and Miscellaneous

HF presents with various underlying causes and culminates in cardiac dysfunction and pump insufficiency, with the most often used experimental animal model of HF being transverse aorta constriction (TAC)-induced pressure overload in mice (Table 4). See et al. investigated single nuclei from CMs in the mouse pressure overload model, in which CMs accurately segregated into clusters specific to sham or TAC groups. Weighted gene co-expression network analysis (WGCNA) revealed a disease module in which the long non-coding RNAs (lncRNAs) *Gas5* and *Sghrt* were identified as key nodal regulators and were correlated with cell cycle genes and the fetal reprogramming marker *Nppa*, providing early evidence for lncRNA-regulated state alteration in stress-response CMs (72). Nomura et al. investigated CMs at various time points after TAC and revealed that activated p53 signaling in the late stage of hypertrophy facilitated the HF gene program (73). They also analyzed single-cardiomyocyte transcriptomes from patients with HF and confirmed the conservation of pathological gene programs. Ren et al. used the iCELL8 platform for scRNA-seq of both CMs and non-CMs in murine hearts after TAC and revealed that fibroblasts underwent a subtype switching away from protective features at the initial stage of hypertrophy. The study also showed that the macrophages switched toward a pro-inflammatory state and that their considerable interaction with CMs was associated with deterioration of cardiac function (74). With the iCELL8 platform, Wang et al. constructed a comprehensive resource of single-cell transcriptomes of both CMs and non-CMs from normal, failed, and recovered human heart biopsy samples. In addition to revealing the inter- and intra-compartmental CM heterogeneity, a major finding of this study was that ACKR1^+^ ECs, which had a protective function and possessed the highest counts of interactions with other cell types, decreased in failed hearts. This observation was validated by *in vivo* experiments where injection of ACKR1^+^ ECs rescued cardiac function after MI by increasing vessel density in both infarct and border regions in mice (10). Zaman et al. demonstrated that cardiac macrophages in the acute and chronic phase after angiotensin II-induced hypertensive stress were enriched in *Igf1*-containing reparative pathways related to wound healing and angiogenesis and that conditional knockout of *Igf1* in tissue-resident macrophages inhibited adaptive CM hypertrophy and led to cardiac dysfunction after long-term hypertensive stress (75). Besides the interaction with CMs, cardiac macrophages have also been reported to activate fibroblasts through paracrine signaling in HF. Ramanujam et al. applied ligand–receptor pair analysis to scRNA-seq data from non-CMs in pressure-overloaded murine hearts by analyzing the transcripts of secreted protein ligands in one cell type and their receptors in another cell type, and predicted that M1 like macrophages interacted with activated fibroblasts *via* secreted proteins such as TGFβ and fibronectin. These interactions were demonstrated to be mediated by macrophage miR-21, depletion of which rescued hypertrophic phenotype after TAC (76). Martini et al., by sequencing cardiac CD45^+^ cells, showed increases in *Osm*^+^ pro-inflammatory macrophages, as well as *Pdcd*1^+^ Tregs, in pressure-overloaded murine hearts when compared with healthy hearts (77).

**Table 4:** Summary of studies of heart failure.

**References**	**Species**	**Model/disease**	**Target tissues/cells**	**Modalities**	**Major findings**
See et al. (72)	Mouse/human	TAC/DCM	Nuclei of CMs isolated from LVs of mice 8 weeks after TAC or sham surgery/end-stage DCM patients or control	snRNA-seq	WGCNA revealed a disease module in which lncRNAs *Gas5* and *Sghrt* were identified as core genes and were correlated with *Nppa, Nppb*, and *Ccng1*. *NPPA* and *NPPB* were also upregulated in human DCM CMs
Nomura et al. (73)	Mouse/human	TAC/DCM	CMs isolated from LVs of mice after sham surgery or 3 days and 1, 2, 4, and 8 weeks after TAC/DCM patients or normal control	scRNA-seq, ChIP-seq	Early hypertrophy was associated with ERK1/2 and NRF1/2 transcriptional networks independent from p53. Activated p53 signaling in late hypertrophy facilitated the heart failure gene program, which was conserved between humans and mice
Ren et al. (74)	Mouse/human	TAC/DCM or HCM	CMs and non-CMs isolated from LVs of mice after sham or 2, 5, 8, and 11 weeks after TAC/end-stage DCM, HCM patients, and control	scRNA-seq	The dynamics of various cell types in the spectrum of heart failure were revealed. Macrophage switching toward a pro-inflammatory state and their considerable interaction with CMs were associated with deterioration of cardiac function, which could be ameliorated by dapagliflozin
Wang et al. (10)	Human	Ischemic HF or DCM	CMs and non-CMs from biopsy samples of LA and LVs of normal, failed and recovered adult human hearts	scRNA-seq	A comprehensive resource of single-cell transcriptomes of both CMs and non-CMs from normal, failed, and recovered human heart was constructed. ACKR1^+^ ECs with a protective function decreased in failed hearts. Injection of ACKR1^+^ ECs rescued cardiac function after MI by increasing vessel density in both infarct and border region in mice
Zaman et al. (75)	Mouse	Angiotensin II	Sorted cardiac macrophages from mice after 4 or 28 days of angiotensin II infusion or sham	scRNA-seq	Cardiac macrophages exposed to hypertensive stress were enriched in reparative pathways encompassing *Igf1*. Either depletion of tissue-resident macrophages or conditional knockout of *Igf1* in tissue-resident macrophages inhibited adaptive CM hypertrophy and thus led to cardiac dysfunction
Ramanujam et al. (76)	Mouse	TAC	Non-CMs from pressure-overloaded hearts 6 days after TAC in wild-type and macrophage-specific miR-21–deficient mice	scRNA-seq	Macrophage-specific deficiency of miR-21 resulted in less fibrosis and attenuated cardiac dysfunction in pressure-overloaded murine hearts. Cardiac macrophages were primary paracrine inducers of fibroblast activation possibly *via* secreted proteins such as TGFβ and fibronectin, which was mediated by macrophage miR-21
Martini et al. (77)	Mouse	TAC	CD45^+^ cells from LVs of mice 1 or 4 weeks after TAC/sham	scRNA-seq	Upon pressure overload, immune activation occurred across the entire range of immune cell types, leading to upregulation of key subset-specific molecules, such as *Osm* in pro-inflammatory macrophages and *Pdcd1* in regulatory T cells

*ChIP-seq, chromatin immunoprecipitation with parallel sequencing; CM, cardiomyocyte; DCM, dilated cardiomyopathy; HCM, hypertrophic cardiomyopathy; HF, heart failure; lncRNA, long non-coding RNA; LA, left atria; LV, left ventricle; scRNA-seq, single-cell RNA sequencing; snRNA-seq, single-nucleus RNA sequencing; TAC, transverse aorta constriction; WGCNA, weighted gene co-expression network analysis*.

Single-cell omics approaches have also played important roles in recent studies investigating SARS-CoV-2–associated cardiac injury. ACE2 (the cellular receptor for virus spike protein) is expressed in CMs and pericytes and is upregulated at both mRNA and protein levels in failing hearts, indicating a vulnerability of these cell states to infection (78, 79). By combining phosphoproteomics and snRNA-seq, Mills et al. reported that the inflammatory response in human cardiac organoids was mediated by STAT1 and epigenetic activation, including BRD4 (bromodomain containing 4, a member of bromodomain and extraterminal protein family). They then validated the results in the hearts of SARS-CoV-2–infected K18-hACE2 mice. Bromodomain and extraterminal family inhibitors were identified as drug candidates that could prevent COVID-19–mediated cardiac damage (80).

## Future Perspectives

Single-cell omics technologies are advancing rapidly and are being applied to a wide range of research subjects. Meanwhile, enabled by progress in bioinformatic analysis, the integration of multi-omics data, as well as spatial transcriptomics, is becoming more common and is providing us with more comprehensive information. These technologies offer deeper insights into the molecular pathways involved in heart development, homeostasis, and specifically in cardiovascular diseases and are thus helping to identify therapeutic candidates.

Besides elucidating pathophysiology, single-cell omics can also contribute to precision medicine. The response to treatment in cardiovascular diseases is non-uniform, as in other organ systems, and this might be associated with interindividual differences in not only genetic backgrounds but also cell populations and microenvironment. Single-cell omics enables assessment of cell populations, functional states, and intercellular communications and thus can enhance patient-specific understanding of diseases alongside the use of existing diagnostic tools. Kim et al. exemplified how single-cell analysis can facilitate a personalized therapeutic approach in a patient with drug-induced hypersensitivity syndrome/drug reaction with eosinophilia and systemic symptoms (81). Such an application to cardiovascular diseases is expected in the near future.

## Author Contributions

ZD: writing—original draft preparation. SN: writing—review and editing, supervision, and funding acquisition. Both authors have approved the submitted version and agreed to be accountable for the content of the work.

## Funding

This work was supported by grants from a Grant-in-Aid for Young Scientists (to SN), the Japan Foundation for Applied Enzymology (to SN), the SENSHIN Medical Research Foundation (to SN), the Kanae Foundation for the Promotion of Medical Science (to SN), MSD Life Science Foundation (to SN), the Tokyo Biomedical Research Foundation (to SN), Astellas Foundation for Research on Metabolic Disorders (to SN), the Novartis Foundation (Japan) for the Promotion of Science (to SN), the Japanese Circulation Society (to SN), the Takeda Science Foundation (to SN), the Cell Science Research Foundation (to SN), a Grant-in-Aid for Scientific Research (B) (to SN), and AMED (JP21ek0210152, JP21gm6210010, JP20bm0704026, JP21ek0210141, JP21ek0109440, JP21ek0109487, JP21gm0810013, JP21km0405209, JP21ek0210118, JP21ek0109406, JP21ek0109543, JP21ek0109569, and JP21tm0724601).

## Conflict of Interest

The authors declare that the research was conducted in the absence of any commercial or financial relationships that could be construed as a potential conflict of interest.

## Publisher's Note

All claims expressed in this article are solely those of the authors and do not necessarily represent those of their affiliated organizations, or those of the publisher, the editors and the reviewers. Any product that may be evaluated in this article, or claim that may be made by its manufacturer, is not guaranteed or endorsed by the publisher.
